# Supplementary material for: Integrative Meta-Analysis: Unveiling Genetic Factors in Meat Sheep Growth and Muscular Development through QTL and Transcriptome Studies
Source: Animals (Basel). 2024 Jun 4;14(11):1679. doi: 10.3390/ani14111679 (PMC11171046; doi:10.3390/ani14111679)
Supplement: Supplementary file 1 [file animals-14-01679-s001.zip › 2.Supplementary File S2.pdf]

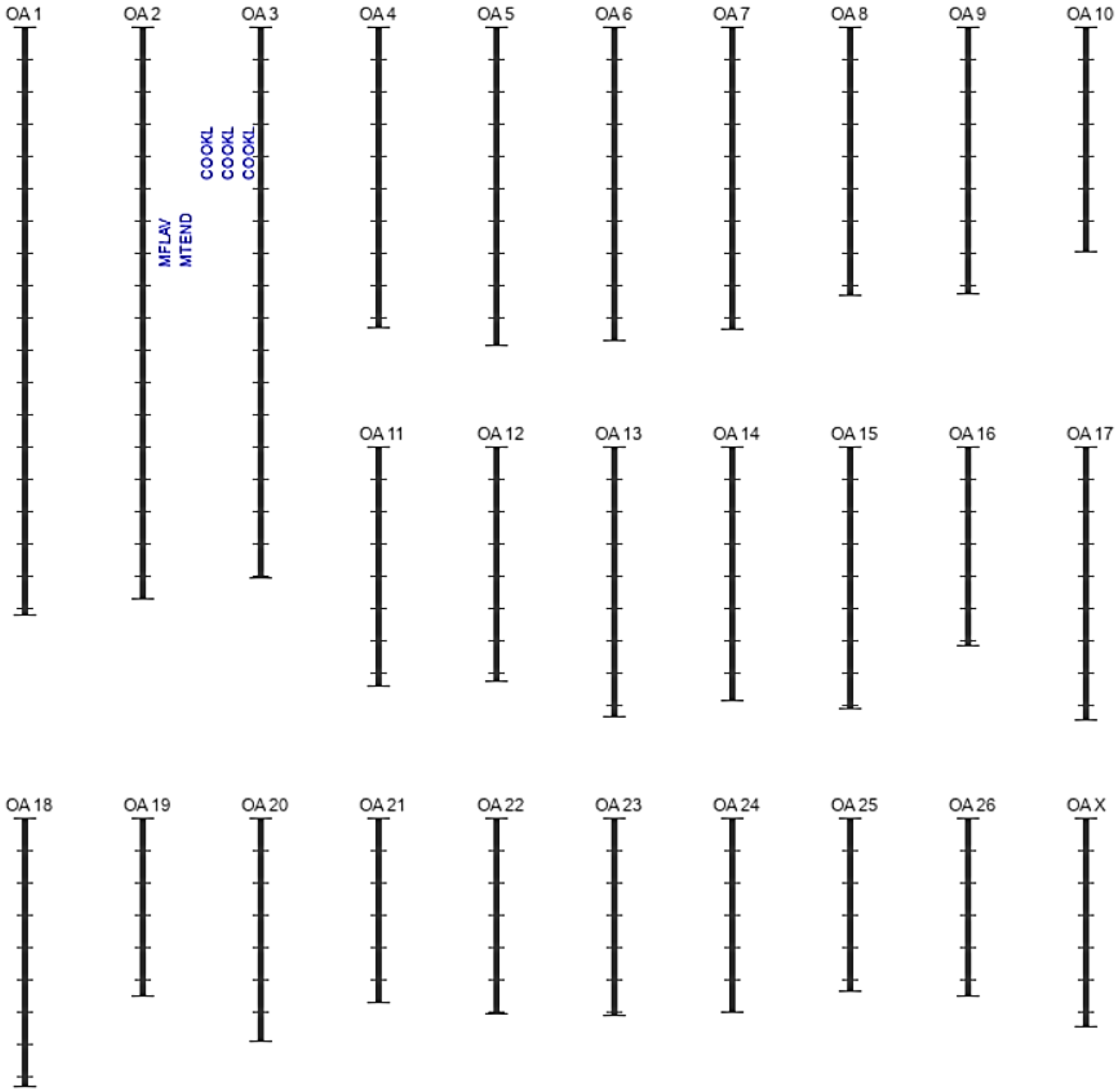

Figure S1. QTL/associations for meat sensory characteristics in the Sheep Genome (It shows genome locations where sensory characteristics traits are mapped by QTL or SNP associations).

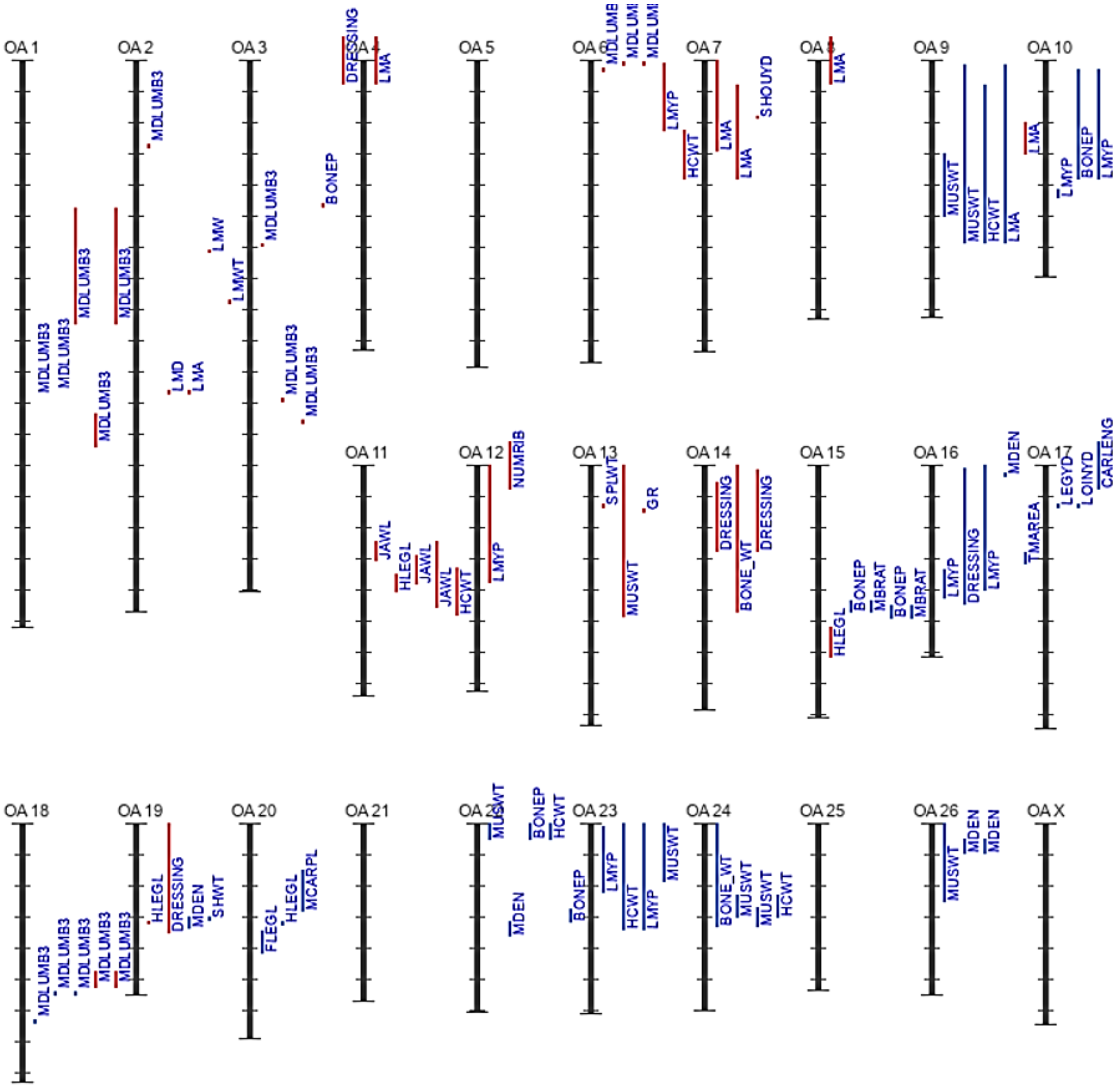

Figure S2. QTL/associations for meat carcass traits (Anatomy) in the Sheep Genome (It shows genome locations where anatomy traits are mapped by QTL or SNP associations).

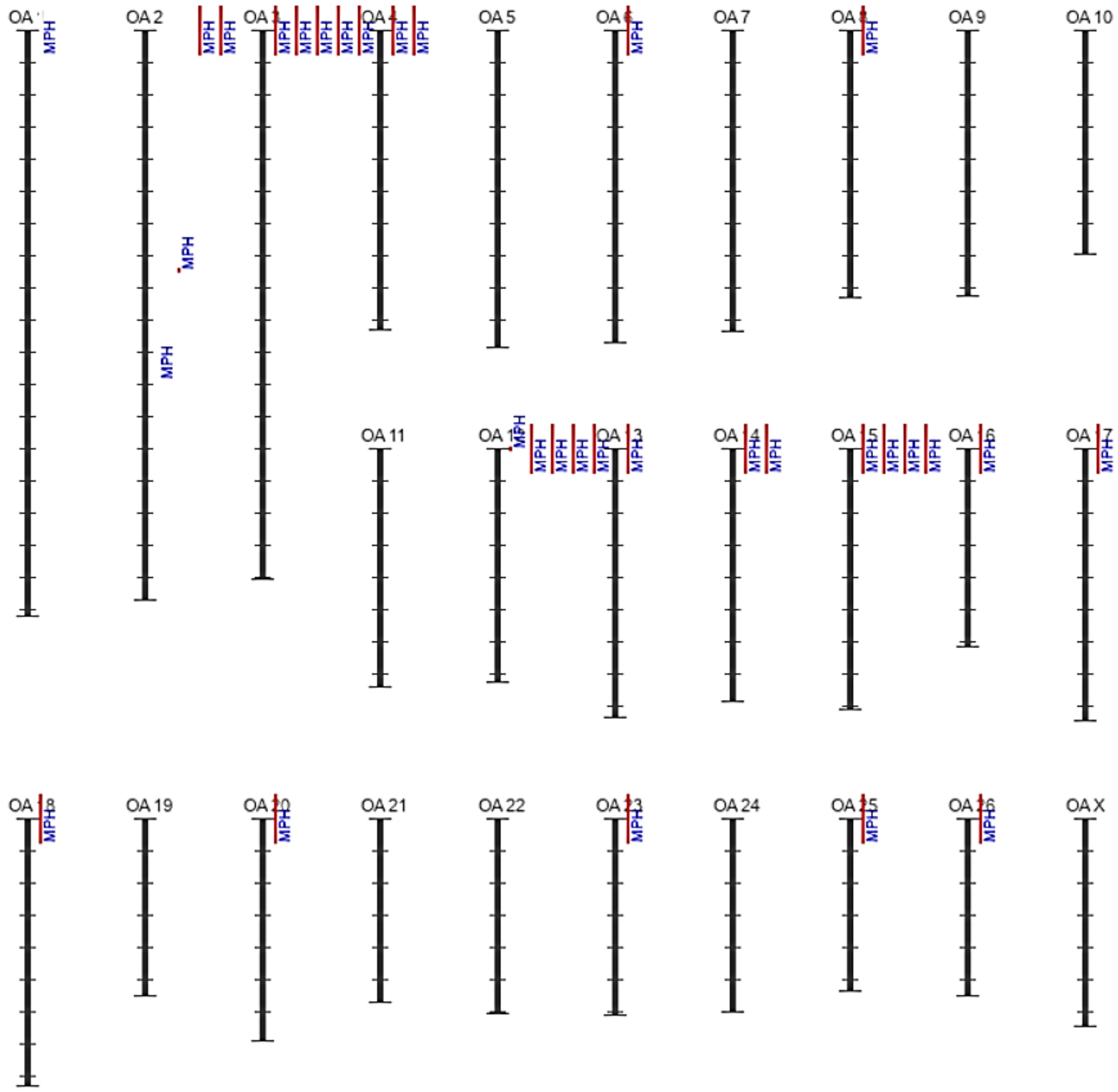

Figure S3. QTL/associations for meat carcass traits (Chemistry/ pH) in the Sheep Genome (It shows genome locations where chemistry traits are mapped by QTL or SNP associations).

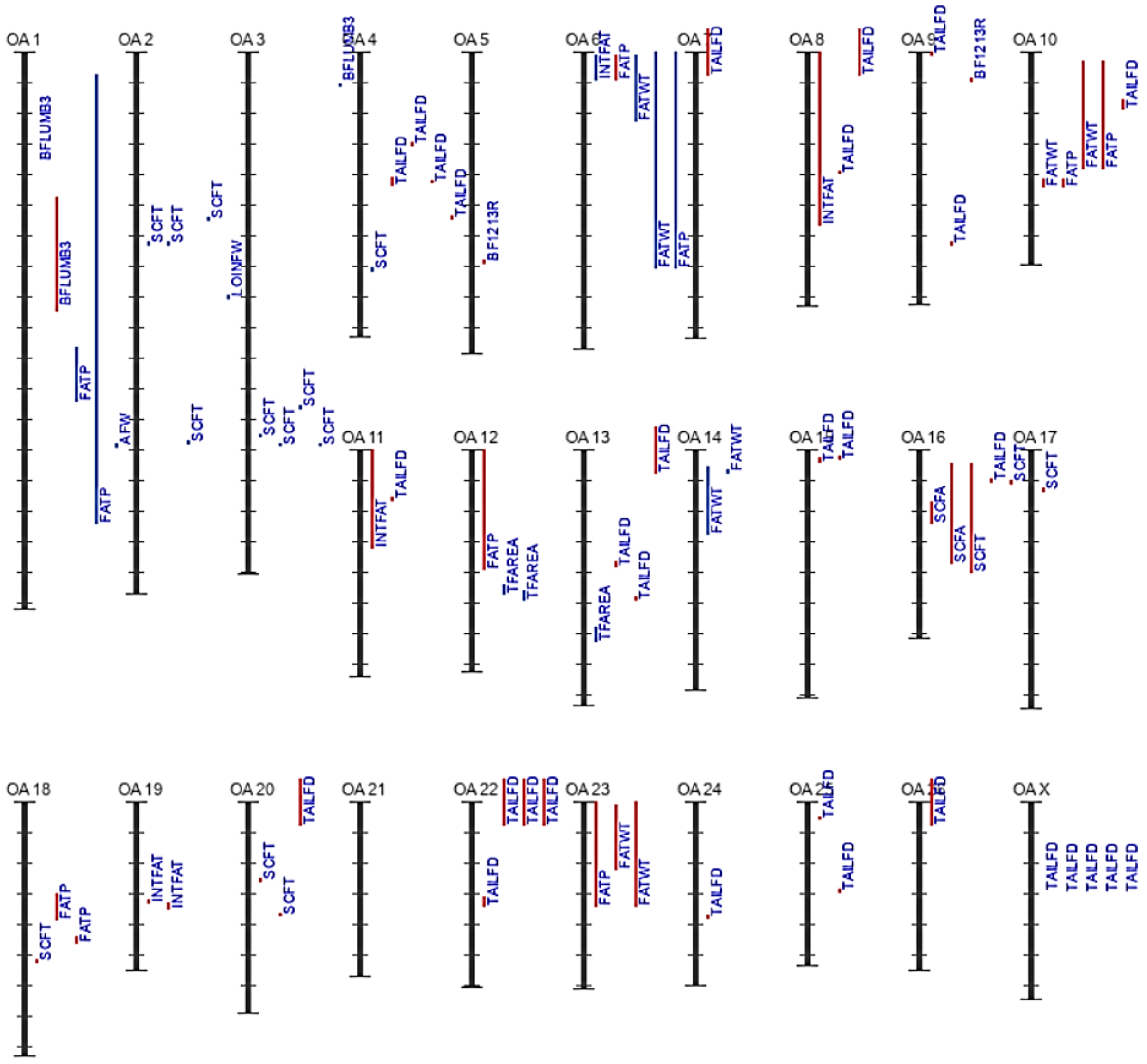

Figure S4. QTL/associations for meat carcass traits (Fatness) in the Sheep Genome (It shows genome locations where fatness traits are mapped by QTL or SNP associations).

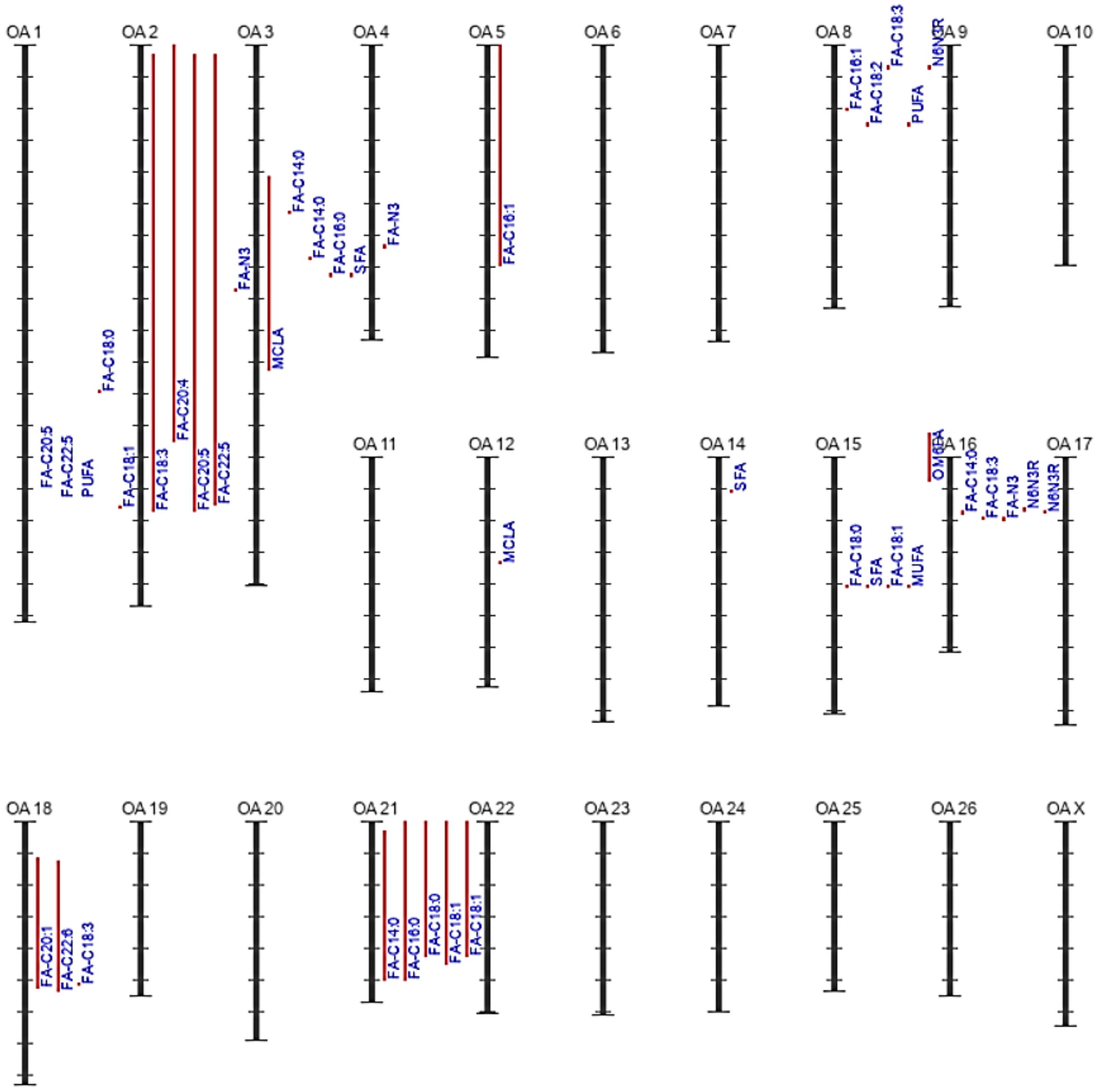

Figure S5. QTL/associations for meat carcass traits (Fat acid content) in the Sheep Genome (It shows genome locations where fat acid traits are mapped by QTL or SNP associations).

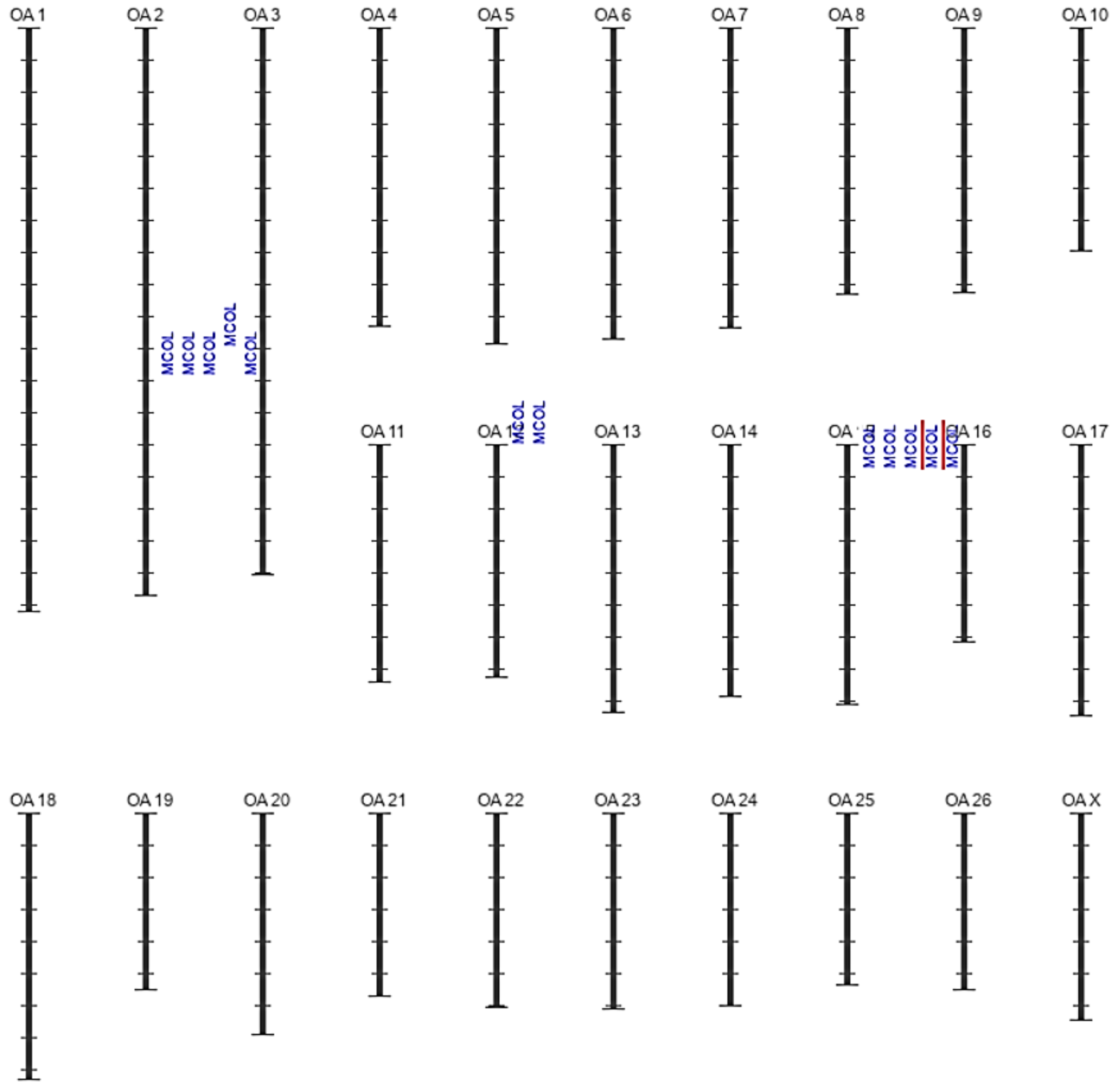

Figure S6. QTL/associations for meat carcass traits (Meat Colour) in the Sheep Genome (It shows genome locations where meat colour are mapped by QTL or SNP associations).

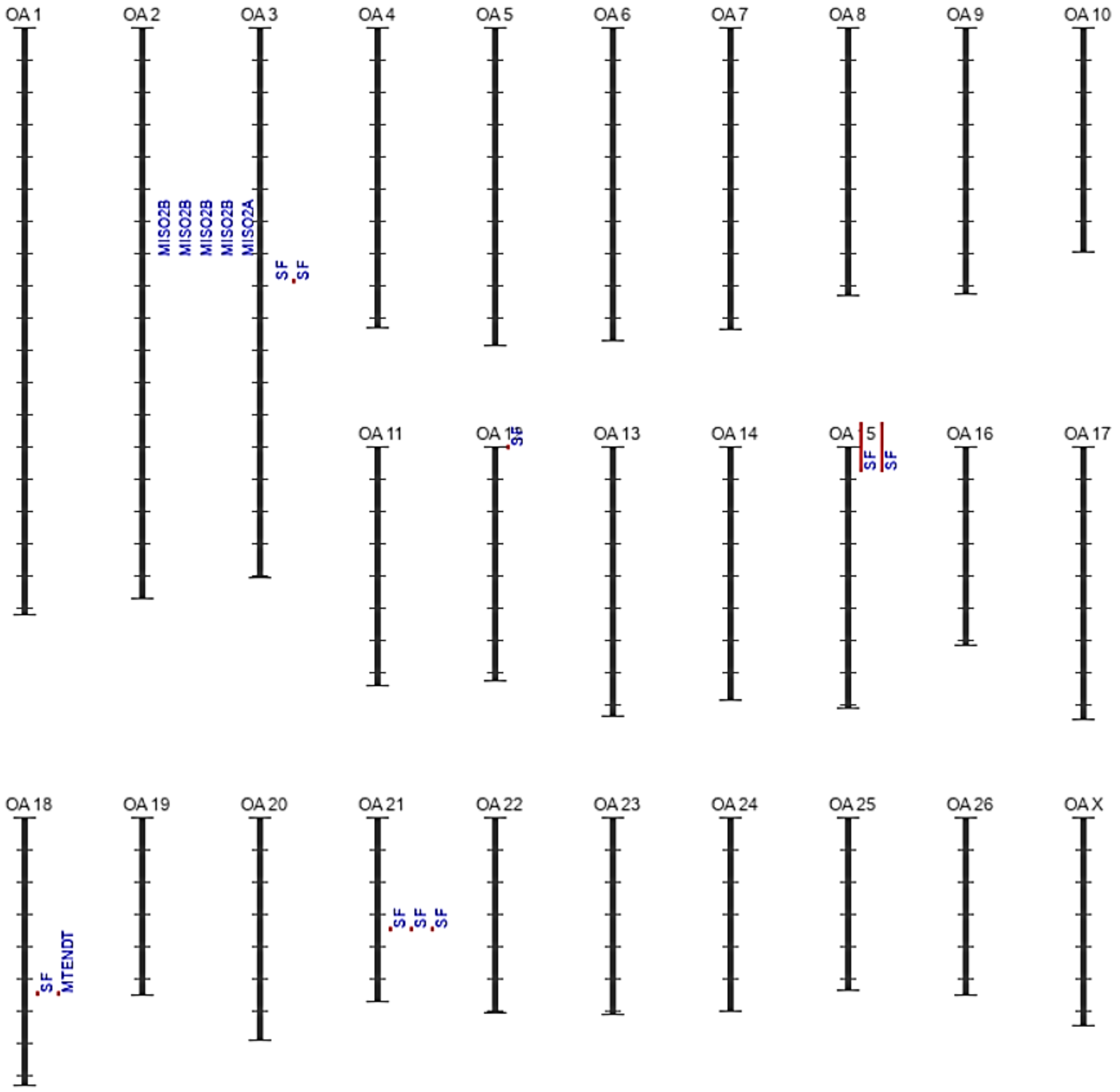

Figure S7. QTL/associations for meat carcass traits (Meat Texture) in the Sheep Genome (It shows genome locations where meat texture traits are mapped by QTL or SNP associations).

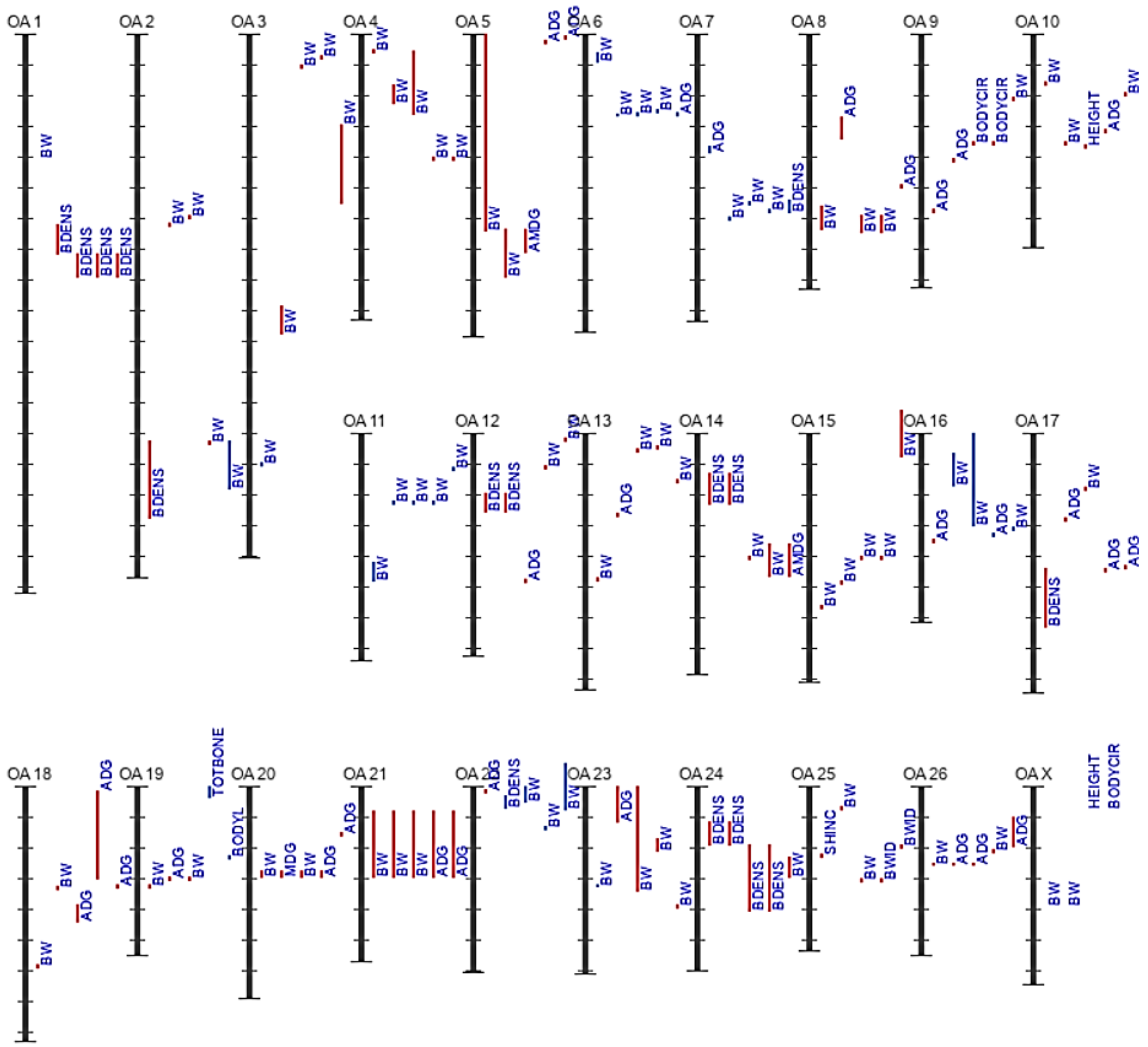

Figure S8. QTL/associations for Growth and carcass characteristics in the Sheep Genome (It shows genome locations where growth traits are mapped by QTL or SNP associations).
